# Correlation between blood pressure control status and cognitive impairment in older adults: A national cross-sectional study

**DOI:** 10.1371/journal.pone.0317861

**Published:** 2025-02-05

**Authors:** Junxiu Yao, Shengshu Wang, Meiling Li, Baomei Song, Cong Lan, Jianjun Jia, Yongjian Yang

**Affiliations:** 1 Department of Cardiovascular Medicine, General Hospital of the Chinese People’s Liberation Army Western Theater, Chengdu, China; 2 Institute of Geriatrics, Beijing Key Laboratory of Aging and Geriatrics, National Clinical Research Center for Geriatrics Diseases, The Second Medical Center, Chinese PLA General Hospital, Beijing, China; Ehime University Graduate School of Medicine, JAPAN

## Abstract

Hypertension is closely related to cognitive impairment; however, the correlation between blood pressure control and cognitive impairment in the hypertensive population is unclear. We aimed to explore the relationship between blood pressure control and cognitive impairment in older adults with hypertension. Using the cross-sectional data from the 2018 Chinese Longitudinal Healthy Longevity Survey, 5,860 people with self-reported history of hypertension were divided into the hypotension, intermediate blood pressure, and hypertension groups. Cognitive impairment was defined as a score of < 18 on the Chinese version of the Mini-Mental State Examination. Demographics of the population were also collected. Bivariate Logistic regression analysis and restricted cubic spline (RCS) model were used to analyze the relationship between blood pressure control and cognitive impairment. After adjusting for confounding factors, binary logistic regression analysis showed that the intermediate group was significantly associated with an increased risk of cognitive impairment than the hypotension group, whereas the hypertension group was significantly correlated with a reduced risk of cognitive impairment. No significant correlation was found between blood pressure control and cognitive impairment in patients aged <80 years, with hearing impairment, or with cerebrovascular diseases, whether in the hypotension or hypertension groups. There was a significant correlation between hypotension and cognitive impairment, and no correlation between hypertension and cognitive impairment in people aged ≥ 80 years, Han ethnicity and those who used antihypertensive drugs. Furthermore, RCS model analysis showed that there was a non-linear relationship between systolic blood pressure and cognitive impairment in individuals aged ≥ 80 years and those using antihypertensive drugs. There was a linear relationship between diastolic blood pressure and cognitive impairment. Blood pressure control in the hypertensive group was significantly associated with a lower risk of cognitive impairment.

## Introduction

Cognitive impairment refers to functional impairment of one or more cognitive domains due to various factors. It has a high prevalence, multiple risk factors, and a complex etiology in older adults. This condition can impact the social function and quality of life of patients to varying degrees and in severe cases, may lead to death [1]. The number of patients with Alzheimer’s disease in China is 15.07 million and is expected to increase to 22.2 million by 2030; moreover, the annual cost of treatment and care for patients with Alzheimer’s disease in China is approximately 670–900 million yuan [2]. Therefore, studying the factors influencing cognitive impairment in older adults is important to reduce the serious burden caused this condition. Hypertension is associated with dementia caused by vascular factors and may promote neurodegenerative diseases in patients with Alzheimer’s disease [3]. Hypertension may lead to a decline in cognitive ability owing to white matter lesions [4,5]. Among the factors that influence cognitive impairment, hypertension, which is a treatable condition, has received increasing attention. Cognitive impairments can be avoided by proper management. Therefore, many studies have focused on the mechanism by which hypertension impairs cognitive function, the time relationship between hypertension and cognitive impairment in the course of life, and the ideal blood pressure goal that should be achieved to minimize the impact of hypertension on the brain [6–8]. Current results on the benefits of lowering blood pressure on cognitive function and possible dementia are uncertain [8]. There is no conclusion on the correlation between blood pressure levels and cognitive impairment in older patients with hypertension. However, the goal of blood pressure management is to reduce the risk of cognitive decline. Herein, we assessed the correlation between blood pressure control and cognitive impairment in older adults with hypertension using data from the 2018 Chinese Longitudinal Healthy Longevity Survey (CLHLS)to provide potential health intervention strategies for the prevention and treatment of cognitive impairment.

## Materials and methods

### Research design and data collection

CLHLS is an ongoing longitudinal study that began in 1998, and conducts follow-up surveys every 2–3 years. It covers 23 provinces, municipalities, and autonomous regions in China, with all data collected through household interviews. This study used data from CLHLS conducted between 2017 and 2018, including general characteristics, lifestyle assessments, ADL assessments, the Mini- Mental State Examination (MMSE), and underlying disease surveys. In total, 15,874 participants were included in the survey. In this study, individuals with a history of self-reported hypertension diagnosis were selected, and those with missing values of the key variables were excluded. Subsequently, 5,860 individuals aged 60–114 years, with an average age of 82.78 years diagnosed with hypertension in the hospital were included; 5,641 (96.3%) and 5,098 individuals (87%) were prescribed antihypertensive drugs. Missing covariate values were filled in using the mode or median. This study was approved by the Ethics Committee of Peking University (IRB00001052-13074). Written informed consent was obtained from all participants or their legal representatives during the survey.

### Variable definition

Relevant variables were collected through structured questionnaires, part of which assessed sociodemographic characteristics, including age, sex, educational background, residence and lifestyle; moreover, health characteristics, including physical activity, measured waist circumference, self-reported diabetes, cardiovascular disease, stroke, other cerebrovascular diseases, respiratory diseases, dyslipidemia, and chronic kidney disease. Age and waist circumference were considered continuous variables, and the others were considered binary variables. The interviewers measured the blood pressure of older adults twice at their residence; the interval between the two measurements was at least 1 min, and the average of the these measurements was recorded as the final blood pressure. Based on clinical experience and previous clinical studies [9], individuals with hypertension were categorized into three groups according to their systolic and diastolic blood pressure levels. Individuals with a systolic blood pressure (SBP) of < 120 mmHg and diastolic blood pressure (DBP) of < 90 mmHg were categorized as the hypotensive group, and those with a SBP of 120–140 mmHg and DBP < 90 mmHg were categorized as the intermediate blood pressure group. Individuals with a SBP ≥ 140 mmHg and/or DBP ≥ 90 mmHg were categorized as the hypertension group.

### Cognitive function assessment

The Chinese version of the Mini-Mental State Examination (CMMSE) was used to measure cognitive function in older individuals using the CLHLS. CMMSE assesses five major aspects: orientation (general ability), response capacity, attention and calculation skills, language and recollection, comprehension and self-coordination skills. It comprises a total of 24 questions. With the exception of seven points for recalling food names within a minute (one point for each, with a maximum of seven points), each question received one point when answered correctly and zero if it was incorrect. The total CMMSE score was 30, with a low score indicating poor cognitive ability. All questions were answered by the interviewees. The CMMSE has been validated in the older Chinese population, and a score of ˂18 indicates cognitive impairment [10,11,12]. In this study, 639 individuals with cognitive impairment were included with a prevalence rate of 10.9%.

### Statistical analysis

The normality test of all continuous variable data was performed, and the data were represented as frequency and percentages. The data from non-normal distribution measurement were represented as quartiles. The chi-square test was used for classified data, the nonparametric test was used for non-normal continuous variables, and binary logistic regression analysis was used to evaluate the correlation between blood pressure control status and cognitive function in the total population and subgroup analyses. A restricted cubic spline (RCS) model with five nodes was used to further analyze the relationships between SBP, DBP, and cognitive impairment. Statistical analysis was performed using SPSS27.0 and Rstudio. A two-tailed test with P < 0.05 was considered significant.

## Results

### Participant baseline characteristics

The characteristics of the study population are listed in Table 1. A total of 5,860 patients with hypertension were included in this study. There were significant differences in blood pressure control status among the three groups in terms of age, ethnicity, marital status, residence, education level, physical labor, past smoking, waist circumference, diabetes, heart disease, cerebrovascular disease, respiratory disease, dyslipidemia, chronic kidney disease, dosage of antihypertensive drugs, cognitive impairment, and ADL scores (Table 1).

**Table 1:** Baseline characteristics of the participants based on blood pressure values.

Variables	Total (n = 5,860)	Intermediate BP group (n = 1,802)	Hypotension group (n = 365)	Hypertension group (n = 3,693)	χ2/z	P-value
Age (years)	82 (75–91)	81 (74–90)	82 (75–92)	82 (75–91)	13.775	0.001
Sex					4.479	0.106
Male	2,523 (43.1%)	812 (45.06%)	158 (43.29%)	1,553 (42.05%)		
Female	3,337 (56.9%)	990 (54.94%)	207 (56.71%)	2,140 (57.95%)		
Ethnicity (Han)	5,656 (96.5%)	1,760 (97.67%)	357 (97.81%)	3,539 (95.83%)	14.12	0.001
Marital status (Married)	2,616 (44.6%)	860 (47.72%)	172 (47.12%)	1,584 (42.89%)	12.416	0.002
Physical exercise (yes)	4,293 (73.3%)	1,188 (65.93%)	251 (68.77%)	2,854 (77.28%)	83.713	<0.001
Waistline (cm)	87 (80–94)	87 (80–93)	85 (78–93)	87 (80–95)	15.271	<0.001
Cognitive impairment, n (%)	638 (10.9%)	185 (10.3%)	61 (16.79%)	392 (10.6%)	13.764	0.001
Hypertension diagnosed in hospital, n (%)	5,641 (96.3%)	1,755 (97.4%)	359 (98.4%)	3,527 (95.5%)	16.725	<0.001
ADL score	6 (6 ~ 6)	6 (6 ~ 6)	6 (6 ~ 7)	6 (6 ~ 6)	9.921	0.007
Living arrangements					7.722	0.021
Independent, n (%)	983 (16.8%)	280 (15.54%)	48 (13.15%)	654 (17.71%)		
Staying with others, n (%)	4,878 (83.2%)	1,522 (84.46%)	317 (86.85%)	3,039 (82.29%)		
BP, mmHg						
SBP	144 (132–159.5)	131 (126–135)	112.5 (108.5–116.5)	155 (146–168)	4,075.77	<0.001
DBP	81 (74–90)	78 (71–81)	68.5 (61.5–74.75)	86 (79–92.5)	1,507.547	<0.001
MABP	102.5 (95–111.5)	95 (90.3–98.2)	82.8 (78–88)	108.7 (103–116.5)	3,265.749	<0.001
Smoking (yes)						
Former	1,662 (28.4%)	542 (30.08%)	118 (32.33%)	1,002 (27.13%)	8.185	0.017
Current	770 (13.1%)	254 (14.1%)	52 (14.25%)	464 (12.56%)	2.905	0.234
Drinking (yes)						
Former	1,408 (24.0%)	435 (24.14%)	94 (25.75%)	879 (23.8%)	0.711	0.701
Current	759 (13.0%)	243 (13.49%)	46 (12.6%)	470 (12.73%)	0.66	0.719
Education (yes)					28.524	<0.001
Literate	2,168 (37.0%)	583 (32.35%)	124 (33.97%)	1,461 (39.56%)		
Illiterate	3,692 (63.0%)	1,219 (67.65%)	241 (66.03%)	2,232 (60.44%)		
Domicile (yes)					228.688	<0.001
City	1,697 (29.0%)	746 (41.4%)	133 (36.44%)	818 (22.15%)		
Villages and towns	4,163 (71.0%)	1,056 (58.6%)	232 (63.56%)	2,875 (77.85%)		
Primary disease (yes)						
Hearing impairment	1,974 (33.7%)	572 (31.74%)	133 (36.44%)	1,269 (34.36%)	5.041	0.08
Diabetes	994 (17.0%)	344 (19.09%)	68 (18.63%)	582 (15.76%)	10.305	0.006
Coronary heart disease	1,449 (24.7%)	530 (29.41%)	111 (30.41%)	808 (21.88%)	43.674	<0.001
Cerebrovascular disease	915 (15.6%)	325 (18.04%)	65 (17.81%)	525 (14.22%)	14.83	0.001
Respiratory disease	579 (9.9%)	201 (11.15%)	47 (12.88%)	331 (8.96%)	10.456	0.005
Dyslipidemia	503 (8.6%)	207 (11.49%)	35 (9.59%)	261 (7.07%)	30.651	<0.001
Chronic nephritis	88 (1.5%)	30 (1.66%)	13 (3.56%)	45 (1.22%)	12.797	0.002
Using antihypertensive drugs					65	<0.001
yes	5,098 (87%)	1,655 (91.8%)	330 (90.4%)	3,113 (84.3%)		
no	762 (13%)	147 (8.2%)	35 (9.6%)	580 (15.7%)		

Data are presented as n (%) or M(P25–P75). BP: blood pressure; SBP: Systolic blood pressure; DBP: Diastolic blood pressure; MABP: mean arterial blood pressure.

### Correlation between blood pressure control status and cognitive impairment in the total population

**Fig 1:**
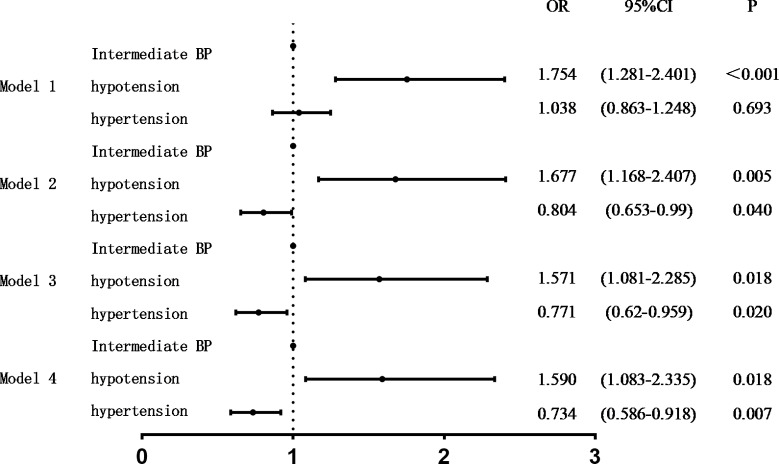
Binary logistic analysis of blood pressure control status and cognitive impairment.

In model 1, univariate analysis showed that compared with the intermediate blood pressure group, there was a significant correlation between the hypotension group and an increase in cognitive impairment (P < 0.001, OR = 1.754), whereas there was no significant correlation between the hypertension group and cognitive impairment (P = 0.693). In Models 2, 3, and 4, compared with the intermediate blood pressure group, there was a significant correlation between the hypotension group and an increase in cognitive impairment (P = 0.005, 0.018, 0.018, OR = 1.677, 1.571, 1.590). Moreover, there was a significant correlation between hypertension and increased cognitive impairment (P = 0.040, 0.020, 0.007; OR = 0.804, 0.771, 0.734) (Table 2 and Fig 1).

**Table 2:** Relationship between blood pressure level and cognitive impairment in the total population.

Model	Independent variable	B	P-value	OR	95%CI	
					Lower limit	Upper limit
Model 1	Intermediate BP group	Reference				
	Hypotension group	0.562	<0.001	1.754	1.281	2.401
	Hypertension group	0.037	0.693	1.038	0.863	1.248
Model 2	Intermediate BP group	Reference				
	Hypotension group	0.517	0.005	1.677	1.168	2.407
	Hypertension group	−0.218	0.040	0.804	0.653	0.990
Model 3	Intermediate BP group	Reference				
	Hypotension group	0.452	0.018	1.571	1.081	2.285
	Hypertension group	−0.260	0.020	0.771	0.620	0.959
Model 4	Intermediate BP group	Reference				
	Hypotension group	0.464	0.018	1.590	1.083	2.335
	Hypertension group	−0.310	0.007	0.734	0.586	0.918

Model 1: Univariate analysis.

Model 2: Adjusted for sex, age, race, marital status, and education.

Model 3: Adjusted for sex, age, race, marital status, education, waistline, physical exercise, living independently, place of residence, history of tobacco and alcohol consumption, and basic daily living abilities.

Model 4: Adjusted for sex, age, race, marital status, education, waistline, physical exercise, living independently, residence, history of smoking and drinking, ability of daily living (ADL), history of antihypertensive drug use, seven primary diseases (hearing impairment, diabetes, heart disease, cerebrovascular disease, respiratory disease, dyslipidemia, and chronic nephritis).

### Hypertension control status and cognitive impairment by subgroup

**Fig 2:**
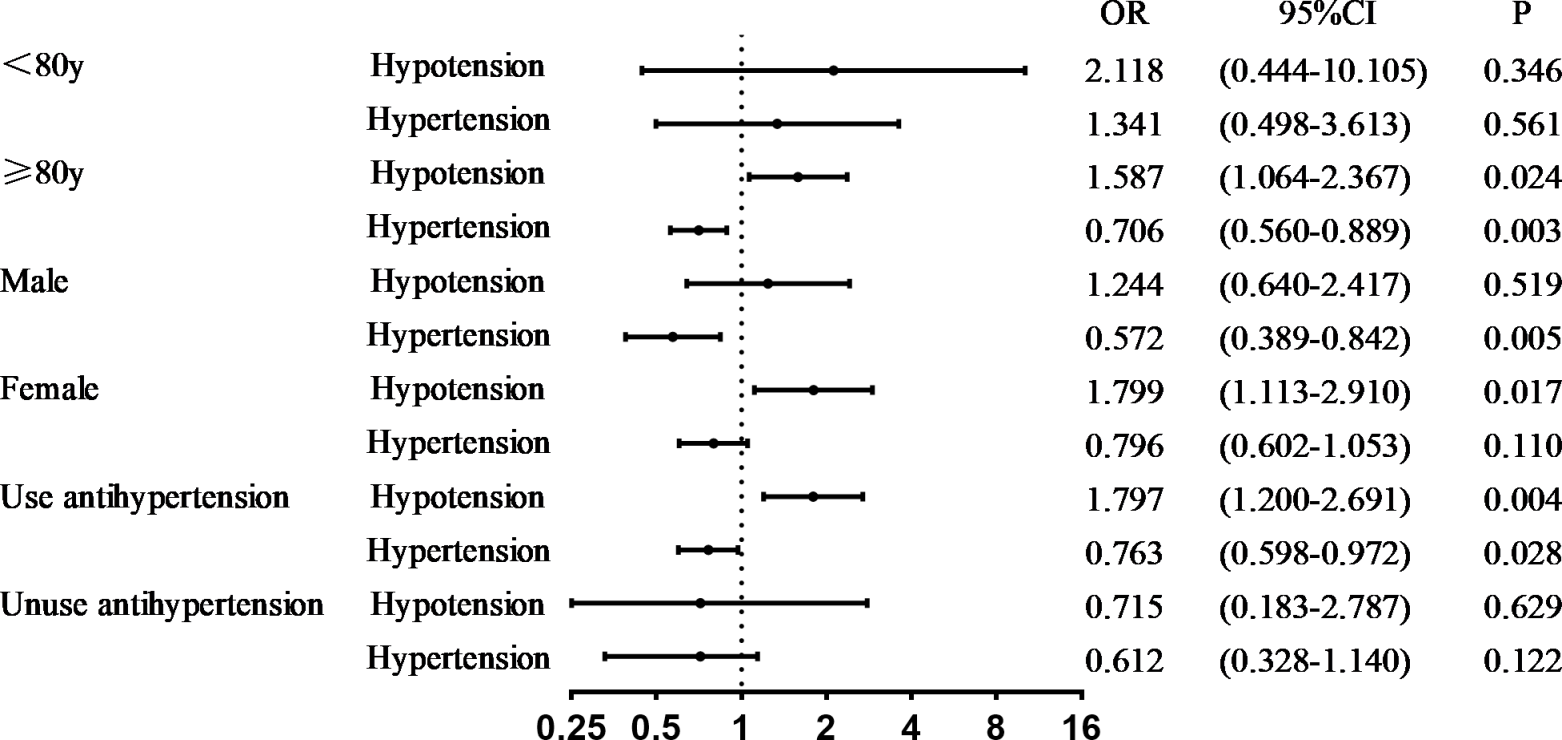
Relationship between hypertension control state and cognitive impairment by subgroup. In the subgroup analysis, the model was adjusted according to age, sex, place of residence, lifestyle, education level, marital status, lifestyle (smoking, drinking, physical activity, waistline), ability to perform daily living (ADL), history of antihypertensive drug use, and seven self-reported diseases (hearing impairment, diabetes, heart disease, cerebrovascular disease, respiratory disease, dyslipidemia, and chronic glomerulonephritis).

In subgroup analysis, compared with intermediate blood pressure, hypotension was significantly correlated with cognitive impairment in people aged ≥ 80 years and those receiving antihypertensive drugs (P < 0.05); moreover, hypertension was not associated with cognitive impairment (P < 0.05). Among women, hypotension was significantly correlated with cognitive impairment (P < 0.05); however, hypertension was not associated with cognitive impairment (P > 0.05). In men, there was no significant correlation between hypotension and cognitive impairment (P > 0.05), whereas hypertension and cognitive impairment were significantly negatively correlated (P < 0.05) (Fig 2).

### Curvilinear correlation between SBP, DBP, and cognitive impairment

**Fig 3:**
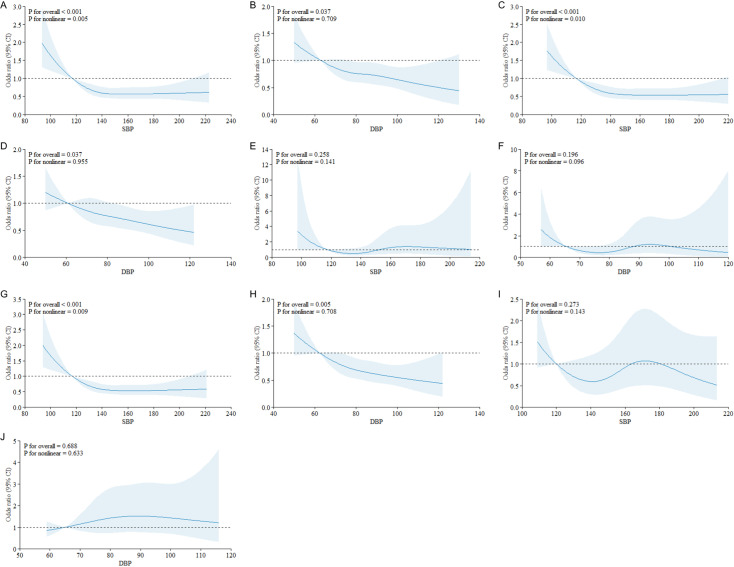
Curvilinear correlation between systolic blood pressure, diastolic blood pressure, and cognitive impairment. Solid lines represent OR values and shadows represent 95%CI.

Using the RCS model to analyze the correlation between SBP, DBP, and cognitive impairment, it can be concluded that in the total population, individuals aged ≥ 80 years, and those using antihypertensive drugs, there is a non-linear relationship between SBP and cognitive impairment after adjusting for sex, age, race, marital status, education, waistline, physical labor, living independently, residence type, history of tobacco and alcohol use, basic daily living, use of antihypertensive drugs, and seven primary diseases (hearing impairment, diabetes, heart disease, cerebrovascular disease, respiratory disease, dyslipidemia, and chronic glomerulonephritis). When SBP was < 140 mmHg, the risk of cognitive impairment increases with a decrease in SBP, whereas, when SBP was > 140 mmHg, the risk of cognitive impairment was relatively stable (P for overall < 0.001; P for nonlinear = 0.005, 0.010, and 0.009, respectively). There was a linear relationship between DBP and cognitive impairment (P for overall = 0.037, 0.037, 0.005, P for nonlinear = 0.709, 0.955, and 0.708, respectively). There was no dose-response relationship between SBP, DBP, and cognitive impairment in people aged < 80 years or in those not receiving antihypertensive drugs (P for overall > 0.05; P for nonlinear > 0.05) (Fig 3).

## Discussion

Hypertension is the main cause of age-related cognitive impairment, which alters the structure and function of cerebral vessels, thus affecting cognitive function [3,13,14]. A community-based cohort study of young adults followed up for up to 30 years revealed that exposure to higher blood pressure levels from youth to middle age was associated with poorer gait and cognitive performance in midlife [15]. In a 32-year prospective follow-up observational study, Smith et al. observed that hypertension in midlife and early late-life significantly increased the risk of dementia; moreover, hypertension assessed at ages 65–74 years has the greatest impact on dementia risk. By 80 years, 15–20% of dementia cases are caused by abnormal blood pressure [7]. In the SPRINT study of ambulatory adults with hypertension, targeting a SBP < 120 mmHg did not significantly reduce the risk of possible dementia than an SBP of < 140 mmHg (primary outcome); however, it significantly reduced the incidence of mild cognitive impairment (MCI), a risk factor for dementia (secondary outcome) [9]. Moreover, in a subgroup of SPRINT participants with a median follow-up of 4.1 years, there was no evidence that intensive SBP control had any effect on memory, language, executive function, or global cognitive function compared with the standard treatment group (targeting 140 mmHg). A slightly larger decrease in processing speed (including the connection test and digit symbol encoding test) was observed in the intensive treatment group, which aimed for a target control level of 120 mmHg, compared to the standard treatment group [16]. A systematic review and meta-analysis suggested that the use of antihypertensive drugs to lower blood pressure is significantly associated with a reduced risk of dementia or cognitive impairment [17]. Another systematic review suggested that there is moderate to low evidence that controlling blood pressure at ≤ 140/85 mmHg does not increase cognitive impairment [18]. [19] A dose-response analysis of five prospective studies of blood pressure and the risk of cognitive impairment and dementia showed that SBP > 130 mmHg in midlife was associated with an increased risk of cognitive impairment; moreover, for blood pressure exposure in later life, increased SBP, low DBP, excessive blood pressure variability, and hypotension are all associated with an increased risk of dementia; Use of antihypertensive drugs reduces the risk of dementia by 21% and a U-shaped dose-response curve shows that DBP levels between 90 and 100 mmHg are associated with a lower risk of developing Alzheimer’s disease.

Multiple prospective observational studies above have reported that blood pressure exposure at high blood pressure levels increases the risk of developing cognitive impairment. Antihypertensive therapy can reduce the incidence of cognitive impairment, and low blood pressure control can increase the incidence of cognitive impairment or lead to a decrease in specific areas of cognitive ability. However, the relationship between blood pressure control and the risk of cognitive impairment in people with hypertension remains unclear. This study used data from a 2018 Chinese national survey, which randomly selected 22 counties and cities in 30 provinces, covering 85% of China’s population. The relationship between blood pressure control status and cognitive impairment in older adults with hypertension was analyzed. After constructing different regression models, it can be concluded that, compared with the intermediate blood pressure group whose SBP was controlled at 120–140 mmHg, the hypotension group with SBP < 120 mmHg was significantly associated with an increased risk of cognitive impairment. This finding is consistent with the previous conclusion that hypotension is associated with an increased risk of dementia. The hypertension group in this study had a blood pressure level of 155 (146–168) mmHg at levels 1–2 of hypertension. The hypertension group with this level of blood pressure was significantly associated with a reduced risk of cognitive impairment. This result is consistent that of another study, which reported a lower risk of developing Alzheimer’s disease when the DBP is between 90 and 100 mmHg (level 1–2–hypertension). The subgroup analysis revealed that among people aged ≥ 80 years with hypertension and those using antihypertensive medications, hypertension was independently associated with a reduced risk of cognitive impairment. This observation is consistent with the results of a study by Smith et al. as well as a systematic review and meta-analysis. In a subgroup analysis of SPRINT participants with a median follow-up of 4.1 years, the intensive blood pressure control group (with a target of less than 120 mmHg) showed a marginally greater decline in processing speed than the standard blood pressure control group. A prospective population cohort study of atherosclerosis risk in a community of 4,761 individuals concluded that patterns of low blood pressure levels in later life were associated with an increased risk of subsequent dementia [20]. A meta-analysis suggested that orthostatic hypotension is associated with an increased risk of dementia and cognitive decline [21]. In the subgroup analysis of this study, hypotension was independently associated with an increased risk of cognitive impairment consistent with the findings of the previous study. Furthermore, the restricted cubic spline analysis showed that there was a non-linear relationship between SBP and cognitive impairment in the general population, people aged ≥ 80 years, and people using antihypertensive drugs after adjusting for confounding factors. When SBP is controlled below 140 mmHg, the risk of cognitive impairment gradually increases with a reduction in SBP. The risk of cognitive impairment remained relatively stable when SBP was > 140 mmHg (P for overall < 0.001, P for nonlinear = 0.005, 0.010, 0.009). There was a linear relationship between DBP and the incidence of cognitive impairment (Overall P = 0.037, 0.037, and 0.005; Nonlinear P = 0.709, 0.955, and 0.708). The results of the restricted cubic spline analysis were consistent with those of the binary logistic regression analysis. A decrease in blood pressure at the end of life may be the result of poor overall health in patients with dementia [22]. However, it remains unclear whether the hypotension observed in our study was due to the overall decline in cardiovascular function in individuals with cognitive impairment at the end of life. Further clarification through a longitudinal cohort study using CLHLS data from follow-up surveys is required. The mechanism underlying hypertension-induced cognitive impairment can be divided into two categories. First, hypertension can directly damage neurovascular units, leading to stroke. Second, it can have indirect effects on the loss of brain autoregulation, neurovascular coupling, amyloid beta accumulation, and microvascular sparsity [23,24]. Increased blood pressure causes increased inflammation and oxidative stress, structural and functional changes in blood vessel, and dysregulation of blood vessels, leading to lesions such as brain small vessel disease and stroke, which, in turn, reduces cognitive function [14,25,26]. When systemic blood pressure is abnormally low or high, impaired brain self-regulation reduces the ability to maintain stable blood flow. In cases of low systemic blood pressure, cerebral blood flow is particularly reduced. Long-term hypoperfusion can reduce brain cell activity, metabolic rate, and brain function. Brain areas related to cognition and brain tissues sensitive to ischemia and hypoxia are in a state of hypoperfusion for a long duration, resulting in delayed neuronal injury and necrosis and reduced cognitive function [20,27]. A study that used observational and genetic data from a large consortium aimed to identify brain structures that may be related to blood pressure values and cognitive function. It combined blood pressure data with 3,935 brain magnetic resonance image-derived phenotypes (IDPs) and cognitive functions, as defined by fluid intelligence scores. The relationship between cognitive function and nine brain magnetic resonance IDPs associated with SBP (including the anterior thalamic radiation, anterior radiative crown, and external sac) was identified by Mendelian randomization analysis, and these structures were analyzed as possible causes of the adverse effects of hypertension on cognitive ability [28].

Hypertension is an important factor affecting the occurrence of cognitive impairment in older adults. In the case of limited treatment for cognitive impairment, it is necessary to actively control hypertension to reduce or delay the occurrence of cognitive impairment and further improve the quality of life of older adults. Most studies on the relationship between hypertension and cognitive impairment have focused on the temporal association between the two throughout the lifespan and the evaluation of treatment duration for hypertension. Relatively few studies have investigated the relationship between blood pressure control and cognitive impairment in older individuals with hypertension. This study involved a wide range of regions and large, diverse population, analyzing the correlation between blood pressure control status and cognitive impairment in older adults of all ages with hypertension in the community. The same conclusion was reached through binary logistic regression and RCS analyses. However, this was a cross-sectional study that can only reflect the correlation between the two; hence, there is insufficient evidence to describe a causal relationship. In addition, this study used only the CMMSE score to assess the existence of cognitive impairment, which lacks imaging evidence. For further validation of our results, cohort studies or randomized controlled trials combined with multidimensional diagnoses and longer follow-up periods are needed.

## Conclusion

In summary, for older patients with hypertension, particularly those aged ≥ 80 years with lower blood pressure control goals were significantly associated with a higher risk of cognitive impairment; Blood pressure control in the hypertensive group was significantly associated with a lower risk of cognitive impairment.
